# Recovery Sleep Immediately after Prolonged Sleep Deprivation Stimulates the Transcription of Integrated Stress Response-Related Genes in the Liver of Male Rats

**DOI:** 10.3390/clockssleep4040048

**Published:** 2022-11-09

**Authors:** Keisuke Fukuoka, Yusuke Murata, Tomomi Otomaru, Masayoshi Mori, Kenji Ohe, Kazunori Mine, Munechika Enjoji

**Affiliations:** 1 Department of Pharmacotherapeutics, Faculty of Pharmaceutical Sciences, Fukuoka University, 8-19-1, Nanakuma, Jonan-ku, Fukuoka 814-0180, Japan; 2 Faculty of Neurology and Psychiatry, BOOCS Clinic Fukuoka, 6F Random Square Bldg., 6-18, Tenya-Machi, Hakata-ku, Fukuoka 812-0025, Japan

**Keywords:** fatigue, integrated stress response (ISR), recovery sleep, sleep deprivation

## Abstract

Sleep loss induces performance impairment and fatigue. The reactivation of human herpesvirus-6, which is related to the phosphorylation of eukaryotic translation initiation factor 2α (eIF2α), is one candidate for use as an objective biomarker of fatigue. Phosphorylated eIF2α is a key regulator in integrated stress response (ISR), an intracellular stress response system. However, the relation between sleep/sleep loss and ISR is unclear. The purpose of the current study was to evaluate the effect of prolonged sleep deprivation and recovery sleep on ISR-related gene expression in rat liver. Eight-week-old male Sprague–Dawley rats were subjected to a 96-hour sleep deprivation using a flowerpot technique. The rats were sacrificed, and the liver was collected immediately or 6 or 72 h after the end of the sleep deprivation. RT-qPCR was used to analyze the expression levels of ISR-related gene transcripts in the rat liver. The transcript levels of the *Atf3*, *Ddit3*, *Hmox-1*, and *Ppp15a1r* genes were markedly increased early in the recovery sleep period after the termination of sleep deprivation. These results indicate that both activation and inactivation of ISRs in the rat liver occur simultaneously in the early phase of recovery sleep.

## 1. Introduction

Sleep loss is one of the most prevalent health problems worldwide. It induces attentional deficit and memory impairment, resulting in increased errors and a reduced ability to detect and correct errors [1,2,3]. Fatigue has been implicated in cognitive dysfunction [4,5,6] and in decreased motivation and depressed mood [7,8,9], both of which are closely related to a lack of sleep. Although aging is a well-known risk factor for fatigue [10], an epidemiological study has shown that children and adolescents also suffer from fatigue and insufficient sleep [11]. In modern society, it is important to focus on the link between fatigue and insufficient sleep to mitigate these negative impacts.

Fatigue is defined as a phenomenon characterized by a decline in physical and mental performance due to the accumulation of physical and mental stress; a fatigued person may require rest or complain of discomfort [12]. Objective and quantitative biomarkers for fatigue have not yet been identified. However, Aoki et al. [13] reported that an increased salivary human herpesvirus-6 (HHV-6) level is a potential candidate biomarker for physiological fatigue, showing that fatigue-induced immunosuppression led to reactivation of HHV-6. Kondo et al. [14] indicated that the reactivation of HHV-6 is associated with the phosphorylation of eukaryotic translation initiation factor 2α (eIF2α). Phosphorylated eIF2α plays a vital role in the cellular stress adaptation system called integrated stress response (ISR), through which cells mitigate the adverse effects of endoplasmic reticulum (ER) stress and oxidative stress by activating the transcription of genes involved in cell survival and repair [15,16,17,18]. Although the relationship between sleep and ISR has not been fully resolved, a recent study has shown that the activation of ISR promotes sleep [19]. Based on the previous findings, it is speculated that the driving of ISR under insufficient sleep conditions is a compensatory response to counteract the accumulation of fatigue due to a lack of sleep. However, to date, the precise relationship between sleep loss and recovery sleep after sleep loss and the ISR remain unclear.

The purpose of this study was to examine the effects of sleep deprivation and restorative sleep on the expression of ISR-related genes through the use of an experimental sleep deprivation technique. We also compared gene transcript expression levels related to cellular stress processing systems other than the ISR, such as ER stress. We selected the liver as the target organ of this study, because several studies have shown that liver is vulnerable to sleep deprivation [20,21,22].

## 2. Results

**Figure 1:**
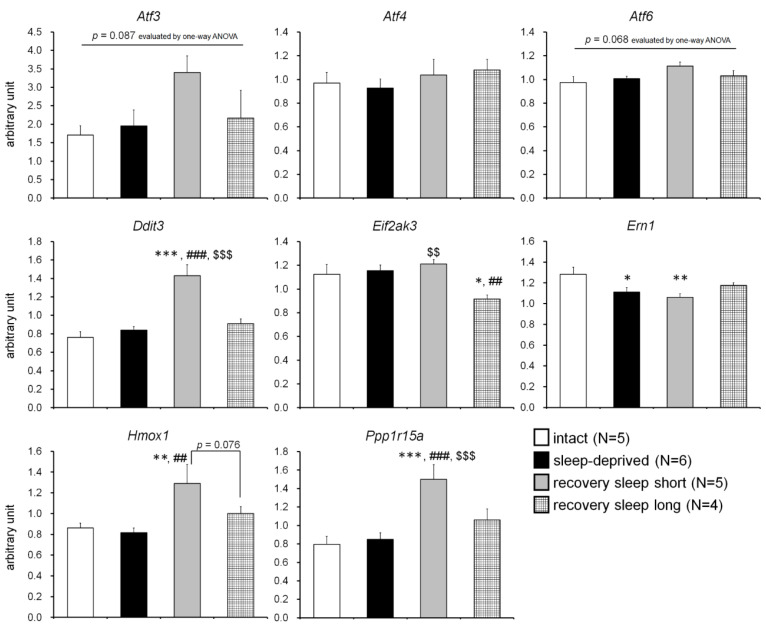
Effects of sleep deprivation and recovery sleep on integrated stress response (ISR)- and unfolded protein response (UPR)-related gene transcript levels of the rat liver. For the ISR-related gene (*Atf3*, *Atf4*, *Ddit3*, *Hmox1*, and *Ppp1r15a*), all transcript levels except for the *Atf4* gene were higher in the recovery sleep short group compared to other three groups. In contrast, sleep deprivation and recovery sleep modestly affected the expression levels of the UPR-related gene (*Atf6*, *Eif2ak3*, and *Ern1*) transcripts. *, **, ***: *p* < 0.05, *p* < 0.01, *p* < 0.001 vs. intact group; ##, ###: *p* < 0.01, *p* < 0.001 vs. sleep-deprived group; $$, $$$: *p* < 0.01, *p* < 0.001 vs. recovery sleep long group.

The data for the relative expression of the target genes of each experimental group are depicted in Figure 1. One-way ANOVA showed significant differences in *Ddit3* (F_3,16_ = 16.917, *p* < 0.001), *Eif2ak3* (F_3,16_ = 4.649, *p* < 0.05), *Ern1* (F_3,16_ = 4.167, *p* < 0.05), *Hmox1* (F_3,16_ = 4.565, *p* < 0.05), and *Ppp1r15a* (F_3,16_ = 8.685, *p* < 0.01). For the *Atf3* and *Atf6* gene transcripts, a non-significant trend toward increased expression was found in the RSS group compared to the intact group (*Atf3*, F_3,16_ = 2.612, *p* = 0.087; *Atf6*, F_3,16_ = 2.884, *p* = 0.068, respectively). No significant difference was found in the *Atf4* mRNA expression levels (F_3,16_ = 0.472, *p* > 0.05). Post-hoc analysis revealed similar changes in the *Ddit3*, *Hmox1*, and *Ppp1r15a* genes, with higher transcript levels in the RSS group compared to the other three groups (*Ddit3*, *p* < 0.001 for intact vs. RSS, SD vs. RSS and RSS vs. RSL, each; *Hmox1*, *p* < 0.01 for intact vs. RSS and SD vs. RSS, *p* = 0.076 for RSS vs. RSL; *Ppp1r15a*, *p* < 0.001 for intact vs. RSS and SD vs. RSS, *p* < 0.05 for RSS vs. RSL). For the *Ern1* gene transcripts, the Bonferroni–Dunn test showed lower expression levels in all other intervention groups compared to the intact group (*p* < 0.05 for intact vs. SD, *p* < 0.01 for intact vs. RSS). In contrast, the *Eif2ak3* expression levels were significantly lower in the RSL group compared to other three groups (*p* < 0.05 for intact vs. RSL, *p* < 0.01 for SD vs. RSL and RSS vs. RSL).

## 3. Discussion

The major finding of this study is that the expression levels of ISR-related genes (*Atf3*, *Ddit3*, *Hmox1*, and *Ppp1r15a*) in the rat liver are markedly increased during the early period of recovery sleep after the termination of sleep deprivation. ISR-related genes are preferentially translated under the induction of ISR in response to cellular stress [23]. ISR is activated by the phosphorylation of eIF2α, which represses general protein synthesis but enhances preferentially transcription/translation of the *Atf4* gene [24,25,26]. Transcription factor ATF4 increases the expression of ATF3 and C/EBP homologous protein (CHOP, the *Ddit3* gene product), which are involved in cell cycle arrest and the induction of apoptosis [27,28,29]. The pro-apoptotic property of ISR leads to stress-exposed cells self-destructing to minimize the negative effects of stress on surrounding cells. In contrast, ATF4 is known to alleviate oxidative stress by enhancing the transcription of heme oxygenase-1 (HO-1, the *Hmox1* gene product), an inducible isoform of heme degrading enzyme [30,31]. Furthermore, the transcription of growth arrest and DNA damage-inducible protein 34 (GADD34, the *Ppp1r15a* gene product), a subunit of eIF2α phosphatase, is activated in an ATF4- and CHOP-dependent manner, which has been shown to inactivate the ISR through a negative feedback loop [32,33]. Based on these findings, the present data of the increased expression of ISR-related genes early in the period of recovery sleep indicate that the activation of ISR and subsequent deactivating responses may be a coping strategy to handle fatigue caused by insufficient sleep. Of interest, the present study found no significant changes in *Atf4* gene transcripts, the key regulator of ISR. This may be due to the short half-life of *Atf4* mRNA (3 h [34]) and the fact that *Atf4* transcription is repressed by the dephosphorylation of eIF2α by GADD34. A limitation of this study is that we only quantified the mRNA expression levels of ISR-related genes. It is well known that the quantification of mRNA levels is not useful for estimating protein levels due to the disregard for translation efficiency [35]. Thus, it will be necessary to evaluate protein expression levels and their phosphorylation status in future studies.

In addition to ISR-related genes, we analyzed the expression levels of genes related to the unfolded protein response (UPR), a cellular stress response similar to the ISR that is involved in the disruption of protein homeostasis, such as normal folding, processing, localization, and degradation of proteins [36,37]. In particular, the accumulation of misfolded proteins generated in the endoplasmic reticulum results in ER stress and triggers apoptosis in cells [38,39]. The major mammalian UPR centers are PKR-like ER kinase (PERK, the *Eif2ak3* gene product), inositol-requiring transmembrane kinase/endoribonuclease 1 (IRE1, the *Ern1* gene product), and ATF6 [40,41,42]. In the present study, differences in the alterations in the expression levels of these UPR-related genes were observed between the experimental groups, but they were not as remarkable as those of the ISR-related genes. Thus, there is a possibility that sleep deprivation and restorative sleep do not significantly affect the UPR in the rat liver. Although previous reports have shown a close relation between sleep and sleep deprivation and ER stress and UPR [43,44,45], most of the basic research has been done in brain regions such as the hypothalamus and cerebral cortex. Pandey et al. [20] evaluated gene transcript expression in the brain and liver of rats treated with 9 days of REM sleep deprivation. In their report, 652 genes were altered in brain and 426 genes were affected in the liver, but only 23 genes had changes in common (10 opposite, 13 in the same directions) across the brain and liver tissue. For example, *WEE1 G2 Checkpoint Kinase (Wee1)*, *Solute Carrier Family 2 Member 12* (*slc2a12)*, and *BCL2 Interacting Protein (Hrk)* genes were commonly downregulated in both the brain and liver. However, the *Hemoglobin Subunit Alpha 1 (Hba-a1)* and *Major urinary protein 5 (Mup5)* genes were upregulated in the brain and downregulated in the liver. *Histocompatibility 2, class II DR alpha (RT1-Da)* and *Zinc Finger and BTB Domain Containing 6 (Zbtb6)* genes were downregulated in the brain and upregulated in the liver. These findings suggest that biological molecular changes induced by sleep and sleep deprivation may be consistent between the central and peripheral regions, while others may not. Indeed, Pandey et al. [20] concluded that the brain functions related to synaptic potentiation, learning and memory, oxidative stress, and circadian rhythms were more vulnerable to REM sleep deprivation. On the other hand, REM sleep loss strongly affected protein synthesis, stress balance, and detoxification in the liver. Of note, Anafi et al. [46] reported that the expression levels of markers of cellular stress and the UPR in peripheral organs (heart and lung) have daily fluctuations. Therefore, sampling times need to be standardized for future studies.

There are several limitations to the present study. First, the sample sizes were small, which necessitates a larger study to confirm our findings. Second, the validation of whether the duration of sleep deprivation and the observational period of recovery sleep was optimal was not adequate. Koban and Swinson [47] reported that the *uncoupling protein-1 (UCP1)* mRNA levels in rat brown adipose tissue were markedly increased as the time of REM sleep deprivation lengthened. In a review of the relation between sleep deprivation and gene expression of several brain areas, the change in expression of immediate early genes, including *c-fos*, *Zif-268*, and *Homer* was dependent on the length of experimental sleep loss [48]. Especially, *brain derived neurotrophic factor (Bdnf)* mRNA levels were unchanged in the rat hippocampus for 6 h of REM sleep deprivation [49] or 8 h of total sleep deprivation [50], but increased in the rat cerebral cortex for 8 h of total sleep deprivation [50,51] and decreased in the rat hippocampus for 8 and 48 h of total sleep deprivation [52]. Interestingly, Guindalini et al. [53] indicated that the expression of *Bdnf* gene transcripts in cerebral cortex was increased after 96 h of REM sleep deprivation, with a return to a normal level after 24 h of recovery sleep. These findings suggest that the duration of experimental sleep deprivation and recovery sleep is critical for evaluating the effects of sleep and sleep loss on gene expression changes in organs. The most significant change in the current study was found in the RSS group, which received 6 h of recovery sleep after 4 days of REM sleep deprivation. At this point, the expression of the genes induced by ISR activation and the genes for deactivation of ISR were concomitantly enhanced, which suggests that the ISR was exclusively activated before 6 h of recovery sleep. Further research with a variable period of recovery sleep will be necessary. Third, the present study did not evaluate the magnitude of the fatigue of our animals. Tanaka et al. [54] reported that a weight-loaded forced swimming test is an appropriate method for the evaluation of fatigue in rats. The platform on the water method was employed to produce sleep deprivation in this study. It is possible that the rats may have become accustomed to immersion after falling asleep. Therefore, it will be necessary to consider the application of spontaneous locomotor activity by the rotating cage or treadmill exercise tolerance to evaluate the state of fatigue due to sleep deprivation. Lastly, the methodology of sleep deprivation in the present study may not be suitable for evaluating the impacts of sleep loss and/or fatigue on ISR-related gene expression. In the flowerpot method, rats are forced to swim in water surrounding a platform and are restricted movement on the platform, which may lead to fatigue and exhaustion. Tanaka et al. [54] established a rat model of sleep loss-related fatigue using a cage with shallow water, in which rats are not forced to swim and are able to move freely. Thus, it will be necessary to compare the methods and select the most appropriate methodology of sleep deprivation to more accurately distinguish sleep loss-related fatigue and nonspecific fatigue. Of note, an intensive study reported by Khalyfa et al. [55] indicated that chronic activation of ISR and inflammation were induced in the visceral white adipose tissue of a murine model of obstructive sleep apnea (OSA). Because OSA caused sleep loss and fatigue [56], it is of importance to clarify the impact of OSA-related sleep loss on the expressions of ISR- and UPR-related genes of rat liver.

## 4. Materials and Methods

### 4.1. Animals

A total of twenty 8-week-old male Sprague-Dawley rats (CLEA Japan Inc., Tokyo, Japan) were kept in groups of 2 or 3 per cage for 1 week after delivery, and then individually housed. The rearing conditions were a room temperature of 23 ± 2 °C, absolute humidity of 60 ± 2%, and a 12-hour light/dark cycle (07:00–19:00: light period, 19:00–07:00: dark period). The rats were provided access to food and water to be consumed ad libitum. Animal experiments were conducted in accordance with the ethical guidelines for animal experiments by the Experimental Animal Care and Use Committee of Fukuoka University (protocol code: 1808046 and date of approval: 1/8/2018), which follows the universal principles of laboratory animal care.

### 4.2. Sleep Deprivation

The sleep deprivation by the flowerpot method was performed according to our previous report [57]. Briefly, the sleep deprivation apparatus consisted of a cubic acrylic tank (30 cm × 30 cm × 30 cm) and an acrylic cylinder (platform, 7 cm in diameter and 10 cm in height). The animals were acclimatized to the apparatus, covered with bedding up to 2 cm below the platform for a consecutive two days. Then, the rats were placed onto the platform in the apparatus, with tap water at 30 °C to 2 cm filled to just below the pedestal. When muscle relaxation associated with REM sleep occurred, the rats could not hold their position on the pedestal, fell into the water, and were awakened (REM sleep-deprived). Access to food and water was permitted ad libitum throughout the experimental period. The water in the apparatus was changed every 24 h.

### 4.3. Experimental Procedure

**Figure 2:**
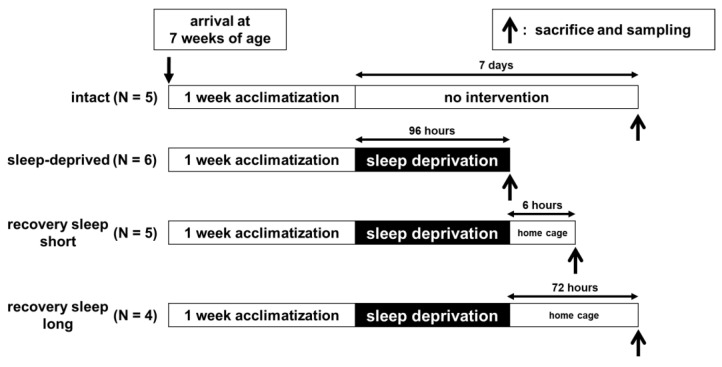
Experimental design. (1) Intact group (N = 5) animals were not subjected to any intervention. (2) Sleep-deprived (SD) group (N = 6) animals were subjected to sleep deprivation for 96 consecutive hours. (3) Recovery sleep short (RSS) group (N = 5) animals were subjected to sleep deprivation for 96 h consecutively, then moved to a normal housing cage to allow them to sleep freely for 6 h. (4) Recovery sleep long (RSL) group (N = 4) animals were subjected to sleep deprivation for 96 hours consecutively, then moved to a normal housing cage to allow them to sleep freely for 72 h. Immediately after the termination of the experimental period (or at a proper timing for the intact group), the animals were decapitated under deep anesthesia and a liver sample was collected to perform RT-qPCR.

The rats were randomly divided into four groups: (1) an intact group (N = 5) was handled daily, but not subjected to any intervention after the start of individual housing; (2) a sleep-deprived (SD) group (N = 6) was subjected to REM-sleep deprivation for a consecutive 4 days; (3) a recovery sleep short (RSS) group (N = 5) was subjected to REM-sleep deprivation for a consecutive 4 days, then moved to a normal housing cage and allowed to sleep freely for six hours; (4) a recovery sleep long (RSL) group (N = 4) was subjected to REM-sleep deprivation for a consecutive 4 days, then moved to a normal housing cage and allowed to sleep freely for a consecutive 3 days. At the end of each experiment, all rats were sacrificed as described below. The experimental schedule for each treatment is shown in Figure 2. 

### 4.4. Sample Collection, Total RNA Preparation, cDNA Synthesis and Quantification of mRNA Level by RT-qPCR

After deep anesthesia with sodium pentobarbital (200 mg/kg, i.p.), each rat was decapitated and a piece of liver (approximately 50 mg) was immediately dissected and immersed in RNAlater (Sigma-Aldrich, St. Louis, MO, USA), then frozen at −80 °C. Total RNA was extracted using Isogen II (Nippon Gene, Tokyo, Japan) and purified with the SV Total RNA Isolation System (Promega, Madison, WI, USA) according to the manufacturer’s protocol with some modifications. Total RNA concentration and A260/A280 ratio were determined using NanodropLite spectrophotometry (Thermo Fisher Scientific, Tokyo, Japan), and the RNA integrity was confirmed using formalin-denaturing agarose gel electrophoresis. Samples with A260/A280 values of 2.0 or higher and sufficient integrity were subjected to subsequent procedures. Next, 0.5 μg of total RNA extracted from liver was reverse transcribed using ReverTra Ace^®^ qPCR RT Master Mix with gDNA Remover (Toyobo Life Science, Osaka, Japan) under the conditions recommended by the supplier. For the quantitative PCR (qPCR) assays, the specific primer oligo DNA for eight target genes (*Atf3* [58], *Atf4* [59], *Atf6* [60], *Ddit3* [60], *Eif2ak3* [60], *Ern1* [60], *Hmox1* [61], and *Ppp1r15a*) and one reference gene (*Actb* [62]) were referred or originally designed. The PCR amplification efficiency calculated by the formula (10^(−1/slope)^ – 1, where the slope was obtained from the regression line of standard curve [63]) was at least 85%. Table 1 shows detailed information on the primer sets. Each qPCR assay was performed using Brilliant III Ultra-Fast SYBR Green^®^ QPCR Master Mix and AriaMx^®^ Real-Time PCR systems (each, Agilent Technologies, Santa Clara, CA, USA). The reaction mixture consisted of 5 µL of 2 × Master Mix, 0.4 µL of primer mix (10 µM each), 3.6 µL of Nuclease-Free Water (Ambion, Waltham, MA, USA), and 1 µL of cDNA. Thermal profiles were as follows: preheating at 95 °C for 3 min; 40 cycles of 95 °C for 10 s and 60 °C for 15 s. A melting curve analysis was subsequently conducted at 95 °C for 30 s, 60 °C for 30 s, and a gradient increase to 95 °C at the rate of 0.5 °C/s, to verify the presence of a single product. To capture intra-assay variability, all real-time qPCR reactions were done in triplicate. To verify non-specific reactions for each primer set, no-template control (water) was included in each assay. The Pfaffl method for the relative quantification of a target gene was performed based on the Cq values that represent the cycle at which the detected fluorescence crossed the threshold, as measured by Aria 1.3 software (Agilent Technologies). In brief, the target gene expression was normalized by the relative quantity of a calibrator (external control, RNA sample extracted from the liver tissue of young male Sprague–Dawley rats independent of the current study). The target gene expression level was corrected by the expression level of a reference gene (*Actb*). The normalized expression was calculated by following formula: (efficiency_target_) ^ΔCq target (external control−sample)^/(efficiency_Actb_) ^ΔCq Actb (external control−sample)^ [64].

### 4.5. Statistical Analysis

One-way factorial analysis of variance (ANOVA) was performed to determine significant differences in the mRNA levels of each gene among the four experimental groups. StatView software Ver.5 (HULINKS, Tokyo, Japan) was used. Shapiro–Wilk and Levene’s tests for the normal distribution of the data and the homogeneity of the variance were verified with R software, version 4.2.1 [65]. If ANOVA showed a significant difference, post-hoc analysis was performed with the Bonferroni–Dunn test. All data are presented as mean ± S.E.M. A *p* value of less than 0.05 was considered statistically significant.

## 5. Conclusions

In conclusion, the present study demonstrated that the expression of ISR-related genes in rat liver is enhanced in the early phase of recovery sleep after sleep deprivation.

## Figures and Tables

**Table 1:** RT-qPCR primers.

Gene Name	GenBank Accession No.	Primer Sequence (5'->3')		Amplicon Size (bp)	Efficiency	R^2^ Value	References
*Actb*	NM_031144	Forward	AAGACAGCACGCTAATAATGC	115	97.84	0.999	[62]
		Reverse	TTGGAAGGCCGGTTAATTTTC				
*Atf3*	NM_012912	Forward	GCACAACATTGGCGTGATTTT	71	97.98	0.999	[58]
		Reverse	TGGCAGACCCCCAAACTCT				
*Atf4*	NM_024403	Forward	TCAGACACCGGCAAGGAG	134	99.75	0.999	[59]
		Reverse	GTGGCCAAAAGCTCATCTG				
*Atf6*	NM_001107196	Forward	TTCTCTGATGGCCGTGCAT	65	93.69	0.999	[60]
		Reverse	TGAAGATGACCCACAGAACCAA				
*Ddit3*	NM_001109986	Forward	TGGCACAGCTTGCTGAAGAG	54	91.09	1	[60]
		Reverse	TCAGGCGCTCGATTTCCT				
*Eif2ak3*	NM_031599	Forward	GGCTGGTGAGGGATGGTAAA	64	86.68	0.998	[60]
		Reverse	TTGGCTGTGTAACTTGTGTCATCA				
*Ern1*	NM_001191926	Forward	GGATGTGAGTGACCGAATAGAAAA	60	89.78	0.997	[60]
		Reverse	TCCAACTGCCGCACGAT				
*Hmox1*	NM_012580	Forward	ACAGCACTACGTAAAGCGTCTCCA	136	89.52	0.998	[61]
		Reverse	CATGGCCTTCTGCGCAATCTTCTT				
*Ppp1r15a*	NM_133546	Forward	CCCAGCATTGTCTACCAGT	84	96.47	0.999	originally designed using Primer3
		Reverse	CAGGTAAATAGAAGGCCACCT				

## Data Availability

The datasets generated and/or analyzed during the current study are available from the corresponding author on reasonable request.
